# Micro-Raman Analysis of Sperm Cells on Glass Slide: Potential Label-Free Assessment of Sperm DNA toward Clinical Applications

**DOI:** 10.3390/bios12111051

**Published:** 2022-11-21

**Authors:** Shengrong Du, Qun Zhang, Haohao Guan, Guannan Chen, Sisi Wang, Yan Sun, Yuling Li, Rong Chen, Youwu He, Zufang Huang

**Affiliations:** 1 The Key Laboratory of Innate Immune Biology of Fujian Province, Biomedical Research Center of South China, College of Life Sciences, Fujian Normal University, Fuzhou 350117, China; 2 Reproductive Center, Fujian Maternity and Child Health Hospital, Fuzhou 350001, China; 3 Key Laboratory of Optoelectronic Science and Technology for Medicine of Ministry of Education, Fujian Provincial Key Laboratory of Photonics Technology, Fujian Normal University, Fuzhou 350007, China; 4 Department of General Surgery, Fujian Medical University Union Hospital, Fuzhou 350001, China

**Keywords:** Raman spectroscopy, sperm DNA, multivariate statistical analysis

## Abstract

Routine assessment of sperm DNA integrity involves the time-consuming and complex process of staining sperm chromatin. Here, we report a Raman spectroscopy method combined with extended multiplicative signal correction (EMSC) for the extraction of characteristic fingerprints of DNA-intact and DNA-damaged sperm cells directly on glass slides. Raman results of sperm cell DNA integrity on glass substrates were validated one-to-one with clinical sperm cell staining. Although the overall Raman spectral pattern showed considerable similarity between DNA-damaged and DNA-intact sperm cells, differences in specific Raman spectral responses were observed. We then employed and compared multivariate statistical analysis based on principal component analysis-linear discriminant analysis (PCA-LDA) and partial least-squares-discriminant analysis (PLS-DA), and the classifications were validated by leave-one-out-cross-validation (LOOCV) and k-fold cross-validation methods. In comparison, the PLS-DA model showed relatively better results in terms of diagnostic sensitivity, specificity, and the classification rate between the sperm DNA damaged group and the DNA intact group. Our results demonstrate the potential of Raman based label-free DNA assessment of sperm cell on glass substrates as a simple method toward clinical applications.

## 1. Introduction

Statistics indicate a nearly 1% per year decline in human reproductive capacity worldwide between 1960 to 2018 [1]. Male factor infertility accounts for approximately 40% of all infertility cases [2]. Semen analysis based on computer-aided sperm analysis (CASA) is well recognized as the most common way to screen semen quality (such as sperm density, concentration, and vitality) and an assistant approach to assessing male fertility [3,4,5]. However, the CASA results are susceptible to being compromised in low and high-concentration specimens, especially for morphological results, due to the heterogeneity between shapes of sperm either in one sample or across multiple samples from one subject [6]. Despite the strict criteria in the latest WHO laboratory manual for normal semen examination and evaluation [7], around 15% of infertile men were still diagnosed with normal semen [8]. By contrast, a positive correlation between sperm DNA fragmentation and reduced male fertility, even impaired offspring fertility, has been confirmed [9], in which the sperm DNA fragmentation index was recognized as a promising detection marker for male infertility diagnosis. More evidence suggests the importance and necessity of evaluating sperm DNA fragmentation along with standard semen analysis [10].

The commonly used staining methods for sperm chromosome assessment include acridine orange (AO) [11,12], sperm chromatin structure determination (SCSA) [13], and sperm chromatin dispersion (SCD) [14]. For instance, the AO test is based on the principle that monomer AO bound to natural DNA emits green fluorescence, while polymerized AO on denatured DNA emits red fluorescence [15], showing high repeatability and inter-assay variability of less than 5%. For SCD, sperm with non-fragmented DNA produces a large halo composed of scattered DNA loops, and the degree of sperm DNA fragmentation can be evaluated based on the presence and size of the halo, in which a halo larger than one-third of the diameter of the sperm head proves intact DNA [16]. Unfortunately, the routine sperm chromatin assessment involves a time-consuming and complex process.

In contrast, Raman spectroscopy characterizes the structure and composition of matter at the molecular level in a label-free, non-destructive manner and coupled with its insensitivity to aqueous backgrounds, it is attractive for biomedical diagnostic applications [17,18,19,20,21]. For example, Raman spectroscopy has been successfully applied to the biochemical characterization of sperm and seminal plasma, as well as Raman spectroscopic imaging assessment of mitochondrial status and DNA damage at the single sperm level [22,23]. However, Raman measurements of sperm cells were usually carried out on a background free substrate to achieve a better signal-to-noise ratio and avoid background interference, in which the metal-plated glass slides, such as aluminum-plated or gold-plated glass, are more favored [24,25]. Additionally, calcium fluoride (CaF_2_), quartz, and fused silica are excellent alternatives [26], but these substrates are undoubtedly more expensive than glass, and impractical for large-scale screening purposes. More importantly, the previous studies reported the averaged Raman spectra of sperm samples in a statistical way, and failed to verify the Raman data of each sperm cell with corresponding staining results in a one-on-one manner. Therefore, cost-effective and disposable glass slides commonly used in optical microscopes offer the possibility of employing the same substrate compatible with sperm staining for Raman measurements and routine diagnostic procedures.

**Scheme 1:**
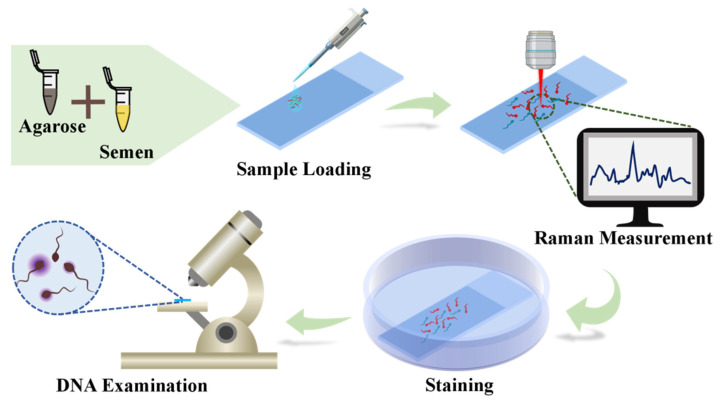
Schematic illustration of label-free Raman measurement of sperm head region and the following staining examination of sperm cells.

As shown in Scheme 1, label-free Raman spectroscopy was performed on sperm smeared on glass slides, and a 532 nm excitation was deliberately selected in combination with the ESMC method to reduce or eliminate signal interference from glass substrates in order to achieve a high signal-to-noise ratio Raman spectrum. Taking the results of sperm DNA staining as a reference, the multivariate analysis based on Raman spectra from DNA-intact and DNA-damaged sperm yielded good results. Preliminary results of the study demonstrate that the Raman spectroscopy with molecular fingerprints validates its feasibility for rapid and label-free differentiation of DNA-damaged and DNA-intact sperm on glass slides.

## 2. Materials and Methods

### 2.1. Reagents and Instruments

Sperm DNA fragmentation staining kits were purchased from Anke Biotechnology Co., Ltd. (Hefei, China). The coverslips (24 × 24 mm) were purchased from CITOTESE Scientific Co., Ltd. (Nanjing, China). 95% ethanol was obtained from Huizheng Pharmaceutical Company (Fuqing, China), and glass Petri dishes (10 cm diameter) were the product of Minbo Co., Ltd. (Fuzhou, China). A Confocal Raman spectrometer equipped with a 532 nm laser (HORIBA, XploRA Plus, Paris, France) was employed for Raman measurement.

### 2.2. Semen Sample Collection and Sperm Preparation

This study was approved by the ethics committee of the Fujian Provincial Maternity and Children’s Hospital, and confirmed that all experiments were performed in accordance with relevant guidelines and regulations. Semen samples were obtained from ten male subjects (age: 36 ± 6) who visited Fujian Provincial Maternity and Children’s Hospital (Fuzhou, China) for a routine fertility test. Informed consent was obtained from all participants in this study. After 3–7 days of sexual abstinence, semen samples were collected by masturbation into a sterile, wide-mouth plastic container and then kept at 37 °C for 30 min to allow liquefaction. For sperm sample preparation, 30 μL of semen was mixed with melted agarose. Next, a volume of 20 μL of semen/agarose mixture was added to the pre-cleaned glass slide and covered with a coverslip, which was then placed in a refrigerator at 4 °C for 5 min to allow the agarose to solidify.

### 2.3. Raman Measurement and Raman Mapping

For Raman measurement, sperm samples on the slide were transferred to the microscope stage, with the coverslip removed, and were examined under a 20× objective to locate the sample area, and then the 100× objective (N.A. = 0.90) was selected to focus the sperm head position. An excitation wavelength of 532 nm with a power of 6 mW was selected to excitate the sperm sample. The Spectrometer grating is 600 gr/mm, with an optimal spectral resolution of 1 cm^−1^. A Raman spectral region of 600 to 1800 cm^−1^ with an exposure time of 20 s and one integration was set for sperm DNA interrogation. A total of 426 sperm cells (361 DNA-intact and 65 DNA-damaged) were measured in this study. Raman mapping of sperm cells was performed to interrogate the spatial distribution of biochemical components in the sperm head. A Raman spectral region of 600 to 1800 cm^−1^ with an exposure time of 1 s and one integration was set. A total of 1634 spectra were measured in an area of 38 × 43 μm^2^, and the resolution of the Raman mapping images is 0.2 μm.

### 2.4. Sperm Chromatin Dispersion (SCD) Test

The glass slide attached with sperm cells was immersed in acid DNA unwinding solution (HCl/deionized water, *v*/*v*, 1:9) for 7 min, and then transferred to 10 mL of lysis solution for 25 min at room temperature. Afterward, the slides were immersed in the deionized water for 5 min, then a 75% ethanol solution for 2 min, and finally in a 95% ethanol solution for 2 min. Next, a certain amount of Wright’s-Giemsa staining solution was added to the air-dried slide and kept for 1–3 min, followed by the addition of an equal amount of phosphate buffer. After 10–15 min, the glass slide was finally washed with deionized water and then dried. Sperm DNA fragmentation was examined by measuring the size of halos under a 50× Raman objective lens, in which the large or medium halos indicated sperms without DNA fragmentation, while small or no halos indicated DNA fragmentation. The DNA staining results were further used to match and verify the corresponding Raman spectra of sperm cells recorded.

### 2.5. Raman Spectra Preprocessing and Multivariate Statistical Analysis

Obtained Raman spectra were first fluorescence background subtracted using the Vancouver Raman algorithm [27], where modified multi-polynomial fitting was employed with a peak-removal procedure, and then followed by spectral smoothing to reduce effects of noise by incorporating a built-in statistical approach. Raman data were vector normalized before further statistical analysis. Origin 2017 software (OriginLab Inc., Northampton, MA, USA) was used to plot the average spectrum, and JMP Pro 16 software (SAS Inc., Cary, NC, USA) was used for a 3D scatter plot of the Raman statistical data. The SPSS13.0 software (SPSS Inc., Chicago, IL, USA) was used for the ROC curve plot and AUC calculation of the two sets of experimental data to evaluate the performance of the classification model.

The extended multiplicative signal correction (EMSC), known for spectral interference subtraction to eliminate known spectral interferences [28], was utilized to remove unwanted background signals. It is based on the idea that a raw spectrum can be described as a linear superposition of the Raman spectrum of interests, the baseline, and the glass signals. Therefore, for the signal correction in our experiment, the Raman spectrum from glass, agarose, and the sperm cells (measured on a metal substrate with no glass and agarose contribution) were obtained, respectively. After baseline correction and normalization, the data set contained all Raman spectra [29] and was then imported into Orange software (Bioinformatics Lab, University of Ljubljana, Ljubljana, Slovenia) to implement the EMSC algorithm.

For statistical data analysis, removing redundant information and extracting meaningful features is essential; therefore, principal component analysis (PCA) [30], a frequently used feature selection method, was first performed to pick the most significant variables based on data reduction manipulation. Instead of directly selecting the largest variance of PCs, 3 PCs (PC1, PC3, PC7, total variance of 36.535%) that are statistically different between groups were utilized after the PCA was complete. Then, linear discriminant analysis (LDA) [31], a supervised approach that discovers new feature subspaces for data projection with maximum separation between classes based on independent variables (wavelengths), was applied. In comparison with PCA-LDA, Partial Least Squares Discriminant Analysis (PLS-DA) [32] is another commonly used supervised method, which provides additional group affinity information by classifying memberships as zeros and ones and, thus can maximize the variations between groups of samples. In particular, PLS-DA highlights the differences among samples from different classes by splitting the hyperspace of the variables and rotating the latent variables (LVs) to achieve maximum group separation, where the predicted response values from Y with a fixed scalar threshold (usually 0.5). PLS-DA coupled with spectroscopic techniques has been successfully applied for qualitative prediction as well as effective discrimination of two groups, especially in the cases of multicollinearity and more variables than observations.

Cross-validation is a process by which the performance of a model is estimated using a limited number of data samples. In this work, two common types of validations, leave-one-out-cross-validation (LOOCV) and k-fold were utilized. In brief, the LOOCV, involves leaving all spectra from a single sample out of the model before assessing performance; while for the k-fold method, a given dataset is randomly split into k equal subsets, where each subset is called as a fold, and one fold was used as the test data set and the other k- 1 folds were used as a training data set.

## 3. Results and Discussion

**Figure 1:**
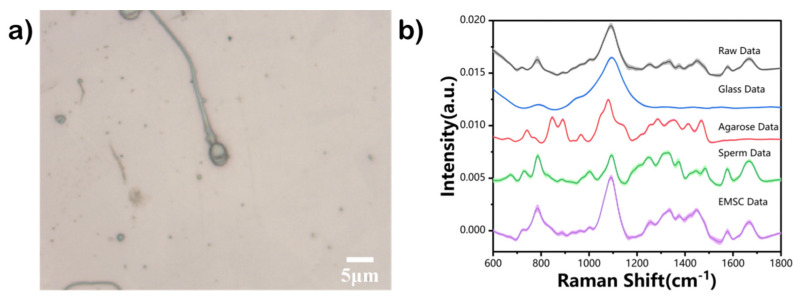
(**a**) Bright-field image of sperm cells on the glass substrate, scale bar = 5 μm; (**b**) The raw Raman spectra of sperm cells (black) obtained from glass slides, Raman spectra of sperm cells (green), and agarose (red) measured on a metal substrate, Raman spectrum of glass slides (blue), and the final background-corrected spectra (purple) obtained by using EMSC algorithm.

Figure 1a shows a representative bright-field image of sperm cells on glass, where the sperm morphological structures (sperm tail, sperm head with acrosome area) can be clearly observed. Figure 1b compares the Raman signals obtained from sperm cell heads and glass substrate, the characteristic Raman bands from the sperm head area on the glass slide, are mainly found in 1200–1800 cm^−1^, consistent with the previous results [33,34]. As expected, a prominent and broad background interference from glass centered at 1050 to 1150 cm^−1^ that overlaps the Raman characteristic peaks from sperm cells was observed. Therefore, it is essential to apply practical methods to reduce background interference and enhance the Raman signals of sperm cells.

EMSC is one of the powerful model-based frameworks that are flexible enough to correct different fluorescence background interferences, and, thus, has been increasingly used in vibrational spectroscopy [35]. Figure 1b shows the removal process of the glass signal from the raw Raman spectrum of sperm recorded on a glass slide using the EMSC method, in which the black line represents the raw spectrum recorded from sperm cells on glass, spectra generated from agarose (red), glass slide (blue), and sperm cells (green) on the metal substrate were set as reference spectra. The purple line is the corrected spectrum, which has had the glass signal subtracted. As expected, by using the EMSC algorithm glass background signal can be effectively reduced.

**Figure 2:**
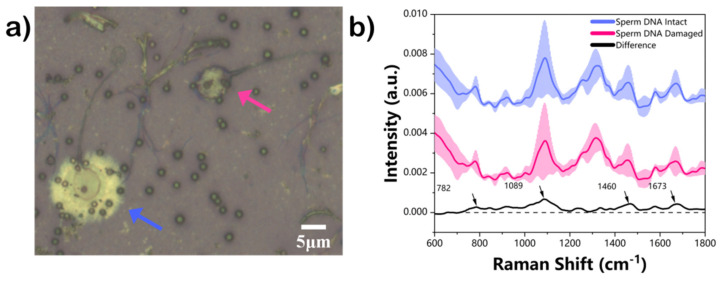
(**a**) Bright-field image of sperm cells on the glass after staining, scale bar =5 μm; (**b**) Raman spectra acquired from sperm cell heads, in which the blue line represents Raman spectra from DNA-intact sperm cell, while the red line indicates the spectra from DNA-damaged sperm cell. The black line represents their difference spectrum.

Previous report links averaged Raman results of sperm cells with DNA fragmentation results such as DFI index in a statistical manner, without knowing the individual sperm DNA status [36]; however, this may potentially degrade the critical yet distinct spectral features that enable the distinguish between sperm cells with damaged and intact DNA. Herein, Raman measurements on sperm cells were performed and followed by immediate staining and examination using the SCD kit. Figure 2a shows a representative staining image of sperm cells on glass slides using the SCD kit, in which sperm heads with large or medium halos (blue arrow) indicate no DNA fragmentation, while small or no halos (red arrow) imply DNA fragmentation. Figure 2b shows the averaged Raman spectral data obtained from DNA-damaged and intact sperm with the DNA staining results as the standard reference. As shown in Figure 2b, relatively higher Raman intensities at 782, 920, 1001, 1089, 1460, 1580, and 1673 cm^−1^ were observed in the sperm DNA-intact group compared with the DNA-damaged group, except for several decreased peaks at 1208, 1258, 1319, and 1367 cm^−1^. The Raman response difference was highlighted in the difference spectrum (DNA-intact minus DNA-damaged) according to the tentative assignment of these Raman peaks, shown in Table 1. Raman peaks at 782, 896, 1089, 1258, and 1319 cm^−1^ were mainly attributed to the DNA of sperm cells. For example, the Raman peak at 782 cm^−1^ has contributions from thymine, uracil, and cytosine vibrations as well as from the DNA backbone. The PO^2−^ backbone shows characteristic vibration around 1089 cm^−1^, the deoxyribose at 896 cm^−1^, the adenine, and cytosine at 1258 cm^−1^, while vibration at 1319 cm^−1^ is mainly assigned to the guanine. Additionally, peaks at 920 cm^−1^ can be assigned to C-C stretch vibration for ribose-phosphate, and the band at 1208 cm^−1^ is assigned to C-C_6_H_5_ vibrations of tryptophan and phenylalanine; and the band at 1460 cm^−1^ was attributed to CH_2_/CH_3_ deformation vibrations for Thymine.

**Figure 3:**
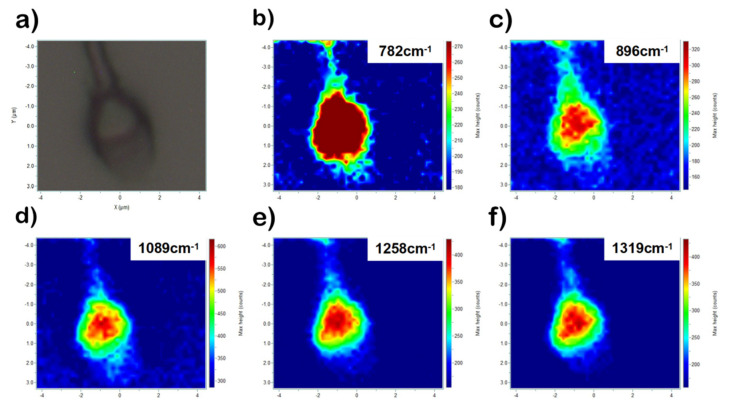
(**a**) Typical morphology of sperm cell head region under a light microscope; (**b**–**f**) Corresponding Raman mapping of sperm head region using band intensities at 782, 896, 1089, 1258, and 1319 cm^−1^, respectively.

Figure 3 compares the Raman mapping of sperm head based on typical Raman peaks assigned to various vibrations of DNA. These mapped bands confirm the correct spatial distribution of DNA content in the sperm head region as well as the robustness of identifying the DNA-rich region in the sperm cell.

**Figure 4:**
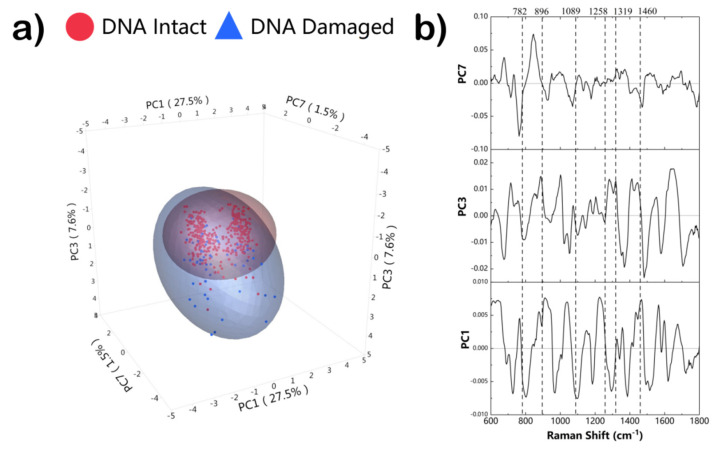
(**a**) Three-dimensional scatter plots of sperm DNA Intact (blue triangle) and DNA damaged (red circle). Ellipsoids represent 95% of data coverage; (**b**) The loading plot shows three (PC1, PC3, and PC7) of the principal components of the sperm Raman spectra.

Although visual inspection of Raman spectroscopy can moderately discriminate between DNA-intact and damaged sperm cells, two multivariate data analysis methods (PCA-LDA and PLS-DA) were used to further evaluate the capability of Raman spectroscopy to discriminate between the sperm DNA-damaged and sperm DNA-intact groups. We selected here three PCs (PC1, 27.5%; PC3, 7.6%; PC7, 1.5%), assessed by independent sample *t*-test, which yield the greatest diagnostic significance for the discrimination of the entire sperm DNA spectrum (*p* < 0.05). Figure 4a shows a 3D scatter plot including the axes of the three primary components from the DNA intact group (361 spectra) and the DNA impaired group (65 spectra), where red triangles and blue circles represent the DNA damaged and DNA intact groups, respectively. As can be seen in Figure 4a, clustered together are the data points of the same group, and mostly separated from the other group. Also, as the enlarged ellipsoid shows, there is an overall heterogeneity within the data of the two groups, revealing to some extent the heterogeneous Raman responses that sperm cells exhibit. Undoubtedly, the reduction of outliers would lead to a smaller radius and less overlap between groups. Furthermore, loadings of the three components (PC1, PC3, and PC7) that significantly contributed to the classification are shown in Figure 4b, where we can explore the vital Raman bands contributing to discriminant analysis. The major bands are comparable to the analyzed sperm spectrum, which confirms the distinction being made is listed. It can be observed that the distinction between DNA intact and DNA damaged sperm is given by the positive and negative values, which correspond to the peaks at ~782, ~896, ~1089, ~1319~1460 cm^−1^, etc. The diagnostic sensitivity and specificity, based on Raman spectroscopy and calculated by PCA-LDA, were 74.8% and 72.3%, respectively. To further enhance the classification results, we also used the PLS-DA algorithm, which adopts the basic principles of PLS and further rotates the components to achieve maximum group separation for better discrimination. Our results showed that the diagnostic sensitivity and specificity calculated by the PLS-DA analysis method for assessing DNA integrity based on Raman spectroscopy were 77.0% and 81.5%, respectively (see Table 2).

Two types of validation schemes, LOOCV and k-fold validation, were used to assess the accuracy of the discrimination models. The PCA-LDA discrimination model correctly classified spectra from DNA intact and DNA damaged groups with accuracies of 74.4% and 73.5% for the LOOCV and k-fold validation schemes, respectively. By contrast, accuracies of 73.5% and 79.8% for PLS-DA model with the LOOCV and k-fold validation were achieved.

**Figure 5:**
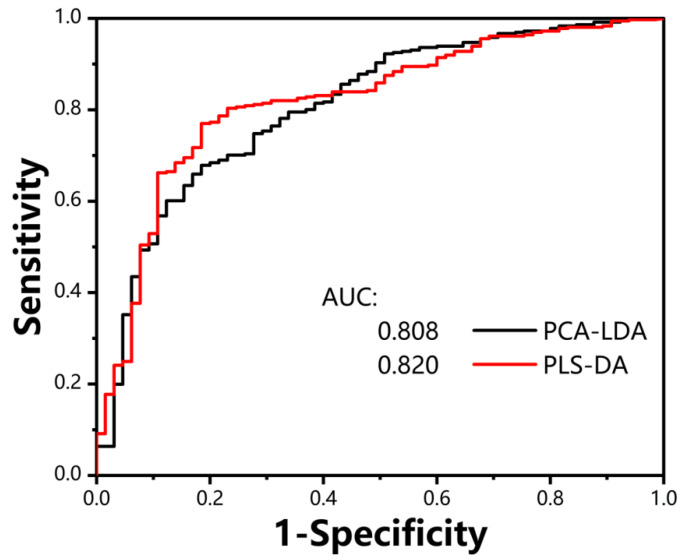
Receiver operating characteristic (ROC) curves for PCA-LDA and PLS-DA for intact and damaged sperm groups.

The receiver operating characteristics curve (ROC) is an important indicator to evaluate the classifier’s performance, as the discrimination threshold is varied. The area under the ROC curve (AUC) serves as an overall measure of the accuracy of the classification model. The higher the AUC value, the better performance of the classification model. Figure 5 compares the differentiation performance between two classifiers of PCA-LDA and PLS-DA. Our calculated AUC values were 0.808 for PC-LDA and 0.82 for PLS-DA, respectively, indicating that the PLS-DA model produced better discrimination between the DNA-intact and DNA-damaged groups.

## 4. Conclusions

In conclusion, this work describes a label-free Raman characterization of sperm cells on a glass slide, and the differentiation of DNA-damaged sperm cells from DNA-intact sperm cells. Although DNA-damaged and DNA-intact groups showed great similarity in the Raman profile, spectral differences can be observed with subtle yet discriminative features. Multivariate statistical data analysis of the Raman spectra of 426 sperm cells showed that compared with the classification accuracy of 74.4% obtained with the PCA-LDA method, the PLS-DA model yields a slightly better classification result of 77.7%. Our results suggest that micro-Raman spectroscopy could potentially be implemented as a reliable and useful tool to distinguish DNA-intact and DNA-damaged sperm cells in a label-free manner. In our future work, we aim to increase the number of samples and include more efficient chemometric methods in order to verify the reliability of the results obtained, as well as to provide in-depth insights into the spectra obtained.

## Figures and Tables

**Table 1:** Vibrational modes and molecular assignment of the Raman peaks.

Wavenumber (cm^−1^)	Vibrational Modes	Molecular Origin
782	Ring breathing modes in the DNA/RNA bases	DNA, thymine, cytosine, uracil
896	C-O-C	Phosphodiester, Deoxyribose
920	C-C	Ribose-phosphate
1001	C-C	Phenylalanine
1089	C-C, PO^2−^ stretch	DNA
1208	υ (C-C_6_H_5_)	Tryptophan, phenylalanine
1258	C-C	Proteins, DNA
1319	CH_3_CH_2_ twisting	Guanine, collagen
1367	υs(CH_3_)	Phosphplipids
1460	CH_2_/CH_3_	Thymine
1580	δ(C=C)	Phenylalanine
1673	C=C	Amide I
1748	C=O	Lipids

**Table 2:** Comparison of discrimination Performance of the DNA-damaged and DNA-intact sperm cells, validated with LOOCV and k-fold method.

Model	Sensitivity	Specificity	Total Accuracy	Validation
PCA-LDA	74.8	72.3	74.4	LOOCV
PLS-DA	77.0	81.5	77.7	
PCA-LDA	73.1	75.4	73.5	k-fold (k = 5)
PLS-DA	78.4	87.7	79.8	

## Data Availability

Data underlying the results presented in this paper are not publicly available at this time but may be obtained from the authors upon reasonable request.
